# Blowpipes and their metalworking applications: New evidence from Mayapán, Yucatán, Mexico

**DOI:** 10.1371/journal.pone.0238885

**Published:** 2020-09-22

**Authors:** Jennifer L. Meanwell, Elizabeth H. Paris, Carlos Peraza Lope, Linda M. Seymour, Admir Masic

**Affiliations:** 1 Center for Materials Research in Archaeology and Ethnology, Massachusetts Institute of Technology, Cambridge, Massachusetts, United States of America; 2 Department of Anthropology and Archaeology, University of Calgary, Calgary, Alberta, Canada; 3 Centro INAH Yucatán, Instituto Nacional de Antropología e Historia, Mérida, Yucatán, Mexico; 4 Department of Civil and Environmental Engineering, Massachusetts Institute of Technology, Cambridge, Massachusetts, United States of America; Utah State University, UNITED STATES

## Abstract

This study presents evidence of two tuyères, or blowpipe tips, used in metalworking at the Postclassic period city of Mayapán. Blowpipe technology has long been hypothesized to be the production technique for introducing oxygen to furnaces during the metal casting process on the basis of ethnohistorical depictions of the process in ancient Mesoamerica. To our knowledge, the tuyères recovered at Mayapán are the first archaeologically documented tuyères for pre-Hispanic Mesoamerica. The dimensions, internal perforation, vitrification, and presence of copper prills within the ceramic fabric, suggest that they were used in pyrotechnological production, likely metalworking, and is consistent with previous evidence for small-scale metalworking at Mayapán. Blowpipe use in metallurgical production is a logical extension of a much longer tradition of blowgun use in hunting, which was likely already present in Mesoamerica by the time metal was introduced to West Mexico from South America. Furthermore, the dimensions of the Mayapán tuyères are consistent with the internal diameter of ethnohistorically-documented blowguns from Jacaltenango in the southwest Maya region. We conducted replication experiments that suggest that when combined with wooden blowpipes, the Mayapán tuyères would have been ideal for small-scale, furnace-based metallurgy, of the type identified at Mayapán from Postclassic period contexts.

## Introduction

Metallurgy is a complex endeavor, requiring mastery of multiple skills, including mining, smelting, pyrotechnology, beekeeping, woodworking, and pottery. As scholarship of ancient Mesoamerican metalworking is increasingly able to ask broader and more nuanced research questions, it becomes possible to identify specific, region-based processes and techniques for groups of producers. Certain aspects of the production process are more archaeologically visible than others, due to their differential preservation. Despite this, numerous studies, including our own, are finding evidence for the production process in evidence from prills [1–3], miscast artifacts [2–4], ingots [3, 5], metallographic sections [1, 6], metallurgical ceramics [4, 7–9], compositional data [1, 3, 5, 6, 10–18], evidence of mining and smelting [19–26], and molds for lost-wax ceramic cores [27].

Metalworking requires high temperature processing and controlled atmospheres for techniques like smelting and casting. The functional parameters of metallurgical ceramics and other production implements are more stringent than for many everyday items used by ancient peoples. Furthermore, the spread of high-skill crafts to new regions means that crafters have to find ways to adapt their existing production techniques, tools, and products to a new crafting industry. The production processes used to create metallurgical tools, such as ceramic crucibles and blowpipes, are influenced by a variety of factors, including performance requirements for the intense heat and the previous knowledge and processes of the artisans who make these tools. Communities of practice can develop, where a group of artisans use consistent production techniques that are learned, shared, and promulgated amongst the community [28–30]. Reconstructing the technical choices made by ancient artisans can be difficult, but detailed studies of production implements and debris can help clarify the technological choices and production steps that characterize a specific tradition, and ideally, begin to reconstruct the operational process or *chaîne opératoire* of production [31–33].

In Mesoamerica, the production of metal objects began in West Mexico by around AD 600 [1, 6, 12] and local metallurgical and metalworking communities of practice also emerged in areas with access to local metal deposits, including central Mexico, Oaxaca, and/or Honduras by the Postclassic period [26, 34]. However, in the Maya Lowlands, karst terrain predominates, and metalworking technologies and metal objects therefore had to be imported over long distances. Mayapán is one of only four Maya sites where evidence of local metallurgical production has been documented [2, 35]. The other three sites were also regional political capitals during the Postclassic period, and include Lamanai in Belize [3, 36, 37], Utatlán in Guatemala [38–41], and El Coyote in Honduras [26, 42].

**Fig 1:**
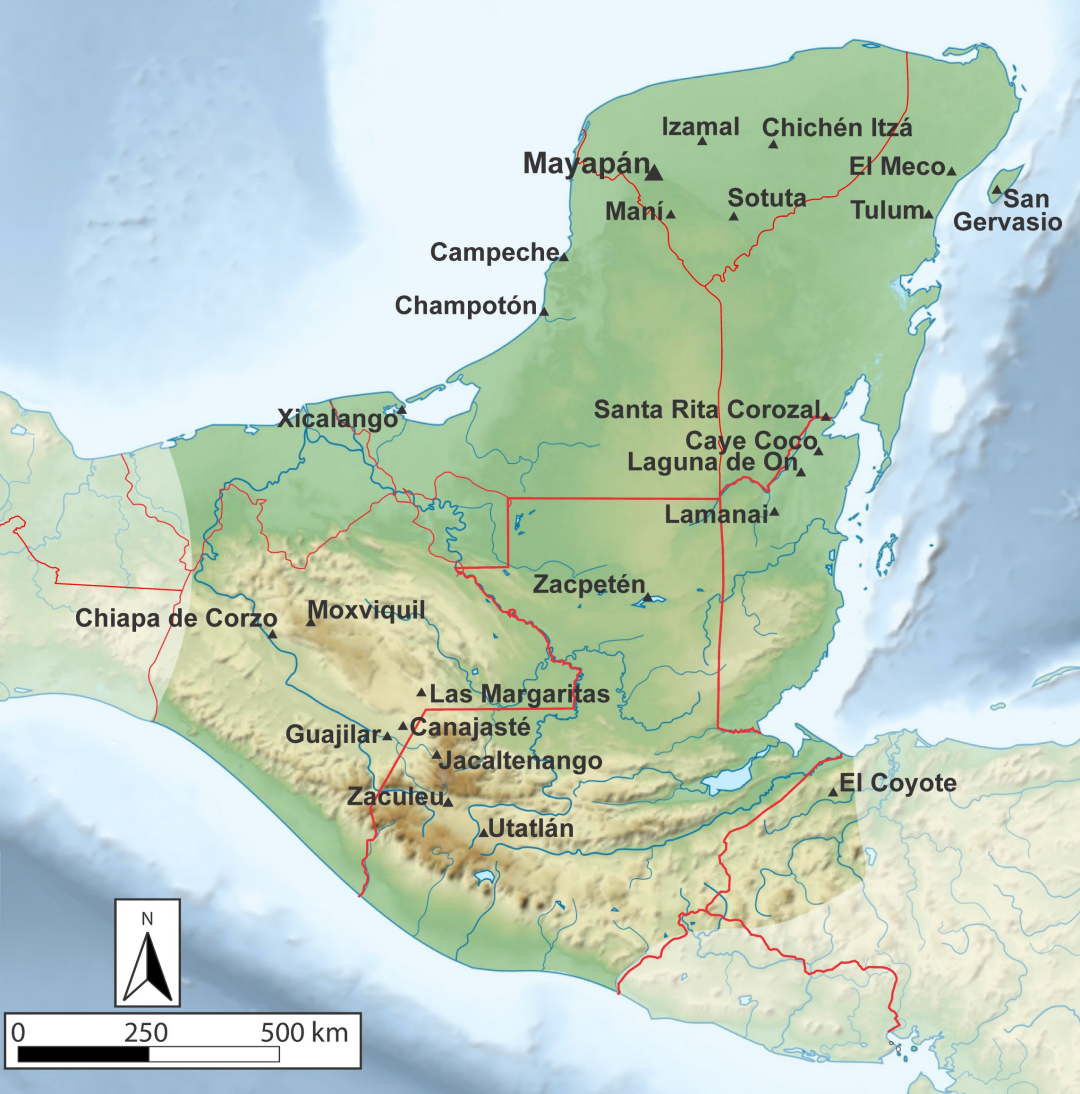
Map of the Maya culture area. Map of the Maya cultural region with sites mentioned in the text. Drafted by Elizabeth Paris, from base map by Wikimedia Commons (©2001 Sémhur/Wikimedia Commons/CC-BY-4.0).

Mayapán was the political capital of northern Yucatán during the Postclassic period (AD 1100–1450), and was an important economic node within an extensive circumpeninsular trade network throughout the peninsula stretching from the Gulf Coast to the Caribbean (Fig 1). During this period, the expansion of commercial networks throughout Mesoamerica created an unprecedented availability of exotic luxury items, raw materials, and high-skill craft technologies at sites throughout the northern Yucatán Peninsula [2, 35]. Mayapán was a center of both local metal production and consumption where residents could obtain metal objects, raw materials, and highly specialized production techniques from distant production zones, despite the differences separating them from sources of metallic ore and a native metalworking tradition [2, 35]. Other raw materials necessary for metalworking, including clay, charcoal, and beeswax from *Melipona beecheii* stingless bees, were local to the Mayapán area and could be adapted to local metalworking applications such as remelting, open-mold, and lost-wax casting [9]. In part, the city was likely able to maintain a local metalworking industry due to its large, diverse, nucleated urban population that was able to support small groups of specialists producing high-value items and regularly attract long-distance merchants [2, 35].

In this study, we identify evidence of blowpipe technology at Mayapán, hypothesized as a means of introducing oxygen to furnaces during metal casting. Although the wooden blowpipes themselves are not known from archaeological examples, we can identify their production through the archaeologically-durable elements used to fabricate and use them, including metallurgical ceramics used as tuyères, or ceramic blowpipe tips. To our knowledge, the tuyères recovered at Mayapán are the first archaeologically documented tuyères for pre-Hispanic Mesoamerica, offering strong, direct evidence for blowpipe use in metallurgy. We further present evidence that Mayapán metalworkers developed their own community of practice, fabricating localized versions of technical ceramics by adapting imported metalworking technologies to their locally-available ceramic resources and blowpipe traditions. The long history of blowpipes as hunting weapons in Mesoamerica was likely one among many points of articulation between different forms of traditional Maya artisanal production and the introduction of metalworking technology to the Maya culture area during the Postclassic period. We analyze two ceramic artifacts using a variety of characterization techniques including petrography, electron microscopy, bulk chemical analysis, and experimental reconstruction. We begin by presenting the context of deposition for the artifacts, explain the analytical methods and experimental data, and discuss the relationship between these artifacts and ethnohistoric accounts of blowpipe use in Mesoamerica. Combined, the evidence suggests that the recovered artifacts were used as blowpipe tips in metallurgical activities at Mayapán. We conclude that Mayapán artisans took advantage of a robust blowpipe tradition and adapted and changed ceramic techniques utilized for pottery vessels to create blowpipe tips ideally suited for local styles of metalworking.

## Materials and methods

### The identification of blowpipe tips from metalworking contexts at Mayapán

Metal artifacts, production debris, and metallurgical ceramics have been found previously in numerous contexts throughout Mayapán’s urban landscape [2, 4, 7–9, 35]. Metal artifacts have been recovered from 59 pre-Hispanic contexts, including structures within and beyond the monumental zone of the site. A total of 559 metal artifacts have been recovered to date, of which a majority are personal ornaments such as small copper bells (N = 482), finger rings (N = 22), tweezers (N = 6), sewing needles (N = 1), miniature axes (N = 1), fishhooks (N = 5), filigree ornaments (N = 1) and sheet metal ornaments (N = 33) [2, 35]. While some of these items were most likely imported from other metal-producing areas in West Mexico, Central Mexico, Oaxaca and Honduras [16], the identification of miscast copper bells [2], crucibles filled with casting debris [2], and metallurgical ceramics [7] strongly support the presence of a local metalworking industry.

A total of 173 metallurgical ceramics have been recovered at Mayapán to date, including metallurgical molds and mold fragments, pestles, and other objects of uncertain function [4, 9]. The ceramic artifacts were first identified by Mayapán project ceramicist Wilberth Cruz Alvarado due to their distinctive dark gray paste and high-fired appearance. The vitrification of these ceramics is visible in petrographic thin sections of two tripod feet from Structure Q-99, hypothesized to be fragments of metallurgical molds [7] and has been confirmed by scanning electron microscopy of the paste (see below).

**Fig 2:**
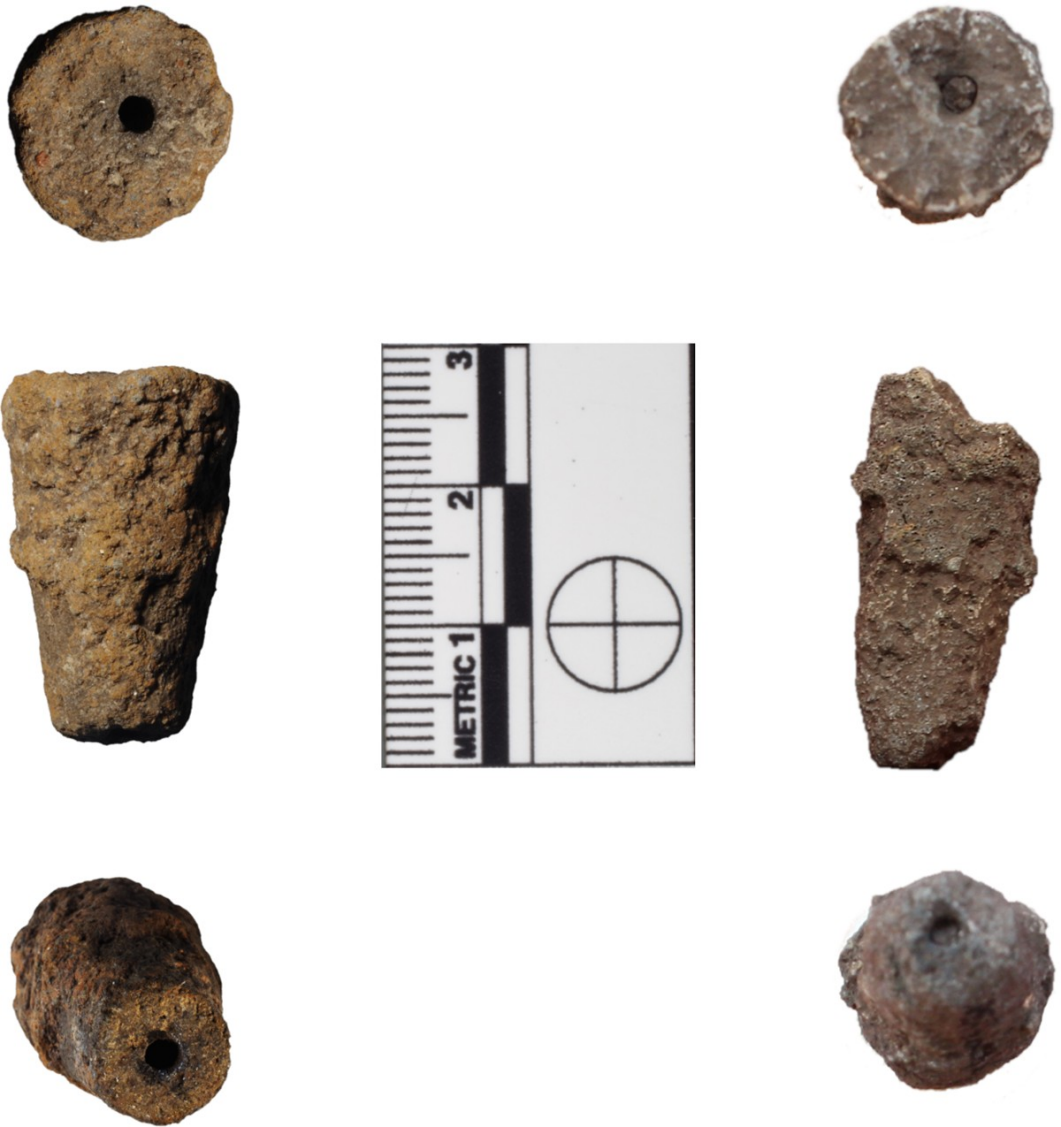
Possible tuyères. Two tapered cylindrical ceramic pieces with hollow channels from Mayapán; we argue that they represent tuyères, or blowpipe tips for metalworking: M-16 (left) and M-55 (right). Composite photographs include proximal, lateral and distal views, from top to bottom. Photograph by Elizabeth Paris and Jennifer Meanwell.

Two of the metallurgical ceramics recovered at Mayapán are tapered cylindrical ceramic pieces with hollow channels running their entire length, which we interpret as tuyères, or blowpipe tips for metalworking (Fig 2). The metallurgical ceramics were excavated and exported by the project Los Fundamentos del Poder Económico de Mayapán, directed by Marilyn Masson, Carlos Peraza Lope, and Timothy Hare. The permit number is 401-36/1687 issued by the Instituto Nacional de Antropología e Historia of Mexico. Like other metallurgical ceramics at Mayapán [7], they are characterized by dark gray, vitrified paste and a lack of macroscopically visible non-plastic inclusions. They are broken at both ends, and would have served to protect the wooden blowpipe from combusting when used to heat charcoal in furnaces or braziers. Experimental studies suggest that the chemical composition of human breath is sufficient to reach temperatures of 1200°C; however, since a cubic meter of human breath generates less heat by combustion than a cubic meter of ambient air, and a human being can maintain blowing only at a certain rate, even small furnaces would have needed several blowpipes operating simultaneously to reach and maintain this temperature [43].

One of the tapered perforated cylinders (M-55) was found in Structure Q-99, while a second tapered perforated cylinder (M-16) was found in Structure Q-40a. Structure Q-99 is a colonnaded hall just to the northeast of the Round Temple in the monumental zone of the site. Structure Q-40a is a small house within the crafts barrio (neighborhood) just to the west of the monumental zone, and is adjacent to one of the largest elite houses in the barrio; it was associated with production implements from numerous craft activities, including molds for figurines and effigy incense burners. Both of these contexts have been hypothesized to be producer contexts, since these two contexts have the largest and second-largest number and variety of metallurgical ceramics associated with them, out of a total of 32 structures associated with metallurgical ceramics at Mayapán. In addition to the similarly high quantity of metallurgical ceramics, the two structures also had similar inventories of types of metallurgical ceramics. Structure Q-99 had a total of 51 metallurgical ceramics, including 4 miniature cup molds, 1 rectangular tripod ingot mold, 1 tapered perforated cylinder, 1 mold fragment, 1 solid cylindrical fragment, and 43 tripod supports. Notably, the metallurgical ceramics were not found in a single area of the structure, but were scattered across 41 different excavated lots (mostly 10 cm levels within 2 x 2 m units). Meanwhile, Structure Q-40a had a total of 33 metallurgical ceramics, including 5 miniature cup molds, 1 mold fragment, 1 tapered perforated cylinder, 1 solid thin cylinder, and 25 tripod supports. As at Q-99, the metallurgical ceramics were recovered from multiple contexts at the structure, distributed across 19 different excavated lots. An additional 9 metallurgical ceramics were recovered at the small neighboring house Q-39, which was also associated with a burial that included 36 metal objects, including copper bells, tweezers, finger rings, and sheet metal ornaments.

### Analytical methods: Archaeological blowpipe tips from Mayapán

A variety of analytical techniques provided data on different aspects of the tapered perforated cylinders from Mayapán. Petrography was chosen to allow a detailed look at the structure and potential inclusions found within the ceramic fabrics. Thin sections were produced using standard techniques and polished. Polished thin sections allowed the use of reflected light microscopy (as is typically performed for metal samples), as well as scanning electron microscopy with energy dispersive X-ray spectroscopy (SEM-EDS) to provide additional insight into the extent of ceramic vitrification and quantitative chemistry on the metal prills. Sample M-16 was sectioned vertically to allow analysis along the perforation, while sample M-55 was sectioned horizontally at two locations to investigate changes between the exterior surfaces and the inner channel. Sample M-16 was made into a polished section to be studied via reflected light microscopy and SEM-EDS. SEM-EDS analysis was performed using a Tescan Vega3 microscope in low vacuum (30 Pa) mode with an accelerating voltage of 20 keV. SEM images were acquired using the backscattered electron (BSE) detector. EDS data were processed and quantified using the Bruker Esprit 2.1 software and linemarker PB-ZAF correction.

### Experimental methods: Identifying functional parameters of blowpipe tips

**Fig 3:**
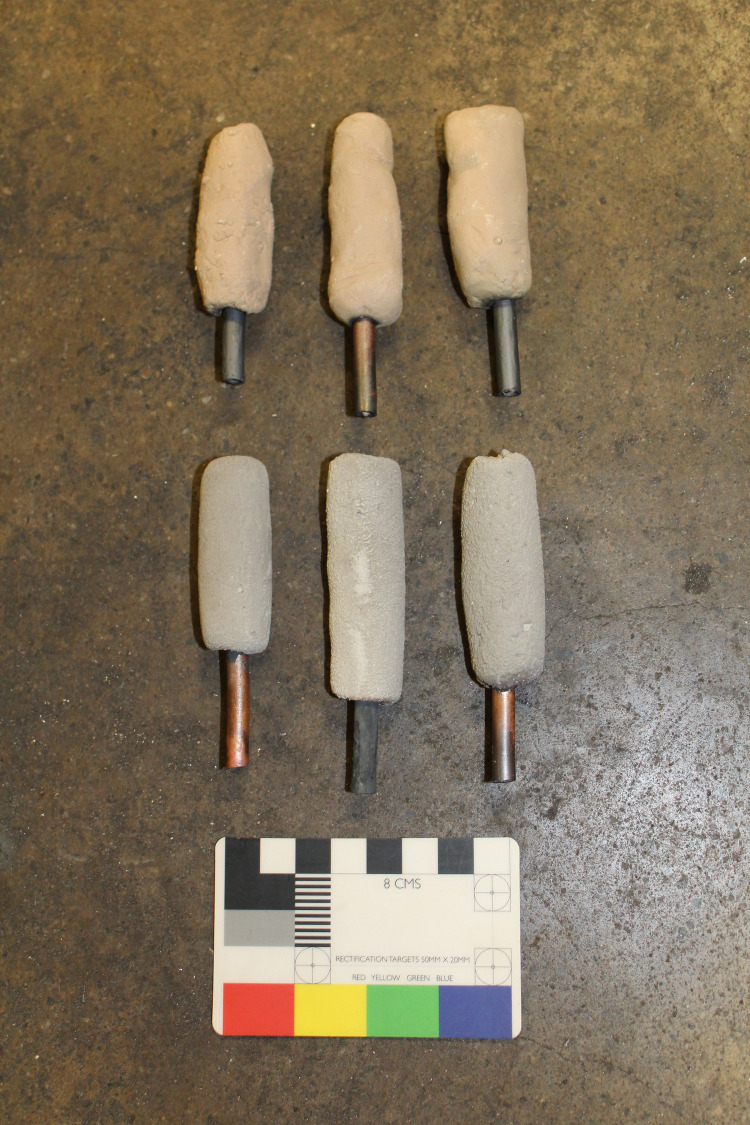
Experimental tuyères. Experimental tuyères made from modern commercial stoneware clay (top row), and local clay from the Mayapán region (bottom row). Photograph by Elizabeth Paris.

As a test of the fundamental parameters for tuyères of the aperture size observed in the Mayapán blowpipe tips, we engaged in a series of replication experiments. For the experiments, Meanwell and Paris prepared experimental tuyères out of two types of clay: modern commercial stoneware clay, and a white clay collected in Yucatán near the village of Mamita, from a road cut along the Mérida-Chetumal highway, approximately 25 km south of Mayapán (Fig 3). The wet clay was fashioned around wood dowels measuring 2 mm in diameter, leaving approximately 4–5 mm in thickness between the dowel and the exterior edge, with overall diameters ranging from 1–2 cm. A copper tube (approximately 4 mm diameter and 5–6 cm long) was inserted into the clay on one end of the tuyère, surrounding the dowel. This tube was meant to make the connections to the air supply easier, but we expect that the actual tips would have been inserted in wooden blowpipes. The tuyères were left to dry in a drying oven overnight, and on the following day, the dowels were removed from the dry tuyères. We then fired the tuyères in an electric kiln. The kiln took just over one hour to go from room temperature to 700°C, and the tuyères were held at temperature for 30 minutes, then slowly cooled over 30 minutes by opening the kiln door slightly. Following the firing, we used the dowels to ensure that the apertures were clear of loose clay that would obstruct them during the experiments.

**Fig 4:**
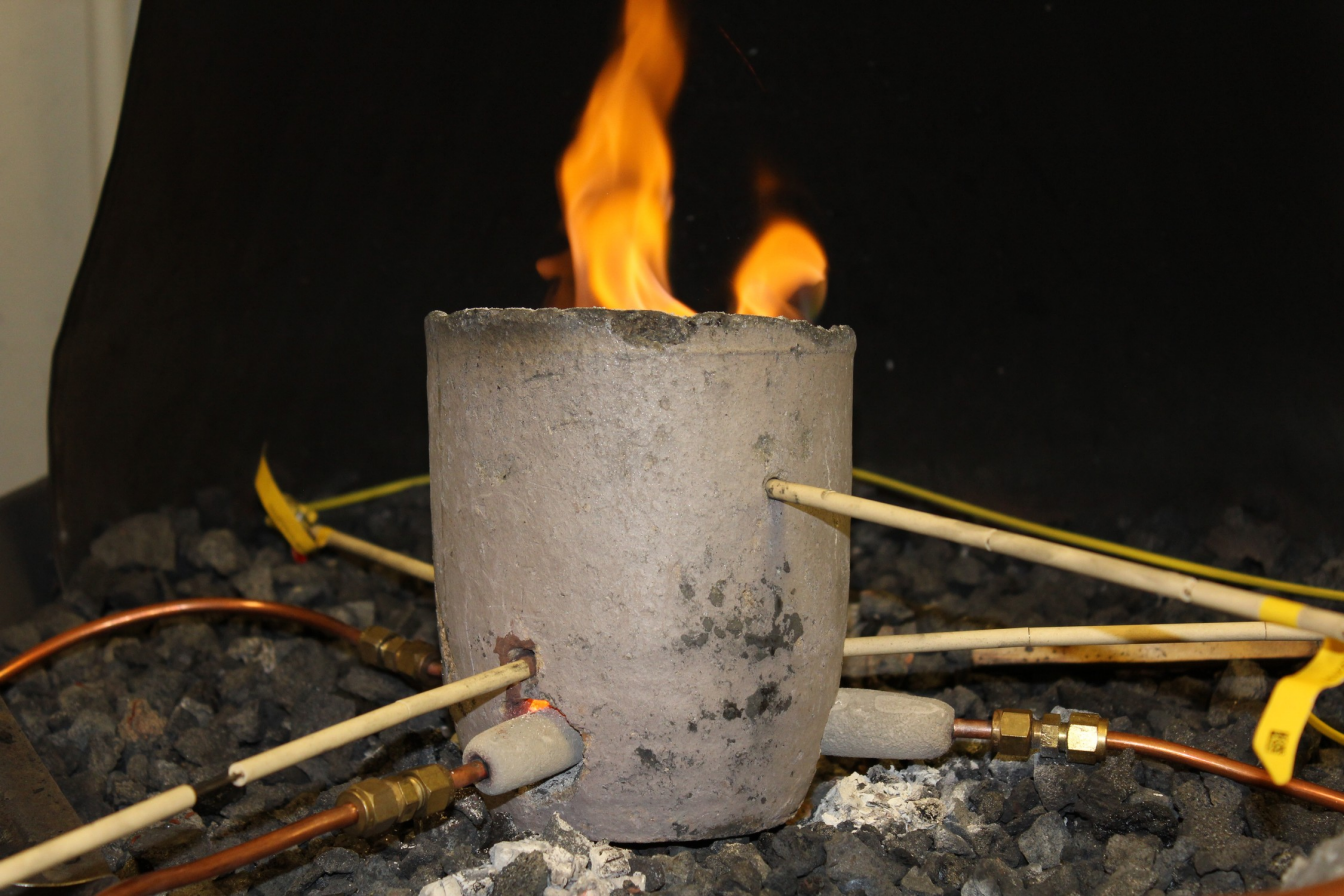
Experimental tuyères and furnace. Experimental tuyère and furnace setup, at the MIT metallurgy forge in the Department of Materials Science and Engineering. Photograph by Elizabeth Paris.

For the experiments, we used the MIT metallurgy forge in the Department of Materials Science and Engineering. We hooked up the experimental tuyères to the forge’s external air supply, filtered through a flow meter, which was set to 2 cubic feet/minute, equivalent to the airflow of a single person. This flow was then split three ways evenly, with constant flow, and a pressure of ~1 psi. Each portion of the split air supply from the flow meter was connected to one of three copper tubes, which were in turn connected to the three tuyères, supplying air to a single stoneware crucible, measuring approximately 15 cm in diameter and 20 cm in height. Holes for the tuyères were drilled approximately 2 cm above the crucible base, with a diameter of 2.5 cm. Three smaller holes were drilled above each of the tuyères for the insertion of a thermocouple, to measure the temperature near the aperture of each tuyère, and a hole for a fourth thermocouple was drilled approximately 2 cm below the rim of the crucible, to measure the internal temperature in the upper portion of the crucible (Fig 4). In the first experiment, the holes around the tuyères and thermocouple for Channel 3 were sealed with Q-set paste (ceramic suspended in acetone), while the others were left open. Temperatures from the thermocouples were recorded using PicoLog 6 software.

For fuel, we used hardwood charcoal purchased from a local grocery store, added over a period of several minutes, and replenished periodically over a period of 20 minutes to fill the crucible to the rim. Although we do not know what species of charcoal would have been most desirable for Mayapán metalworkers, lime kiln replication experiments suggest that there are six species of trees that are particularly sought after due to their hardness and density: *chacah* (*Bursera simaruba*), *jabin* (*Piscidia piscipula*), *chucum* (*Pithecellobium ibicans*), *tzalam* (*Lysiloma latisiliquum*), *katzin* (*Acacia gaumeri*), and *tzitzilche* (*Gymnopodium antigonoides*) [44]. These species are readily available in the area immediately surrounding the archaeological site of Mayapán. Russell and Dahlin [44] note that some woods like mahogany, cedar, and flamboyant were specifically avoided by experienced local lime kiln builders at Mayapán, because they do not burn all the way through, or for other, unstated reasons. Many of the traits that produced desirable results for lime production may also have been desirable for heating crucibles for metalworking. During the first experiment, the charcoal was added in various sized chunks, directly from the bag. For the second experiment, the charcoal was crushed to a more consistent size of approximately 2–3 cm in diameter before being added to the crucible. After the replica tuyères were used in the firing experiments, they were made into polished thin sections, like the archaeological samples, so the microstructure and vitrification present in each paste could be evaluated using petrography and SEM.

## Results

### Petrographic analysis and reflected light microscopy

**Fig 5:**
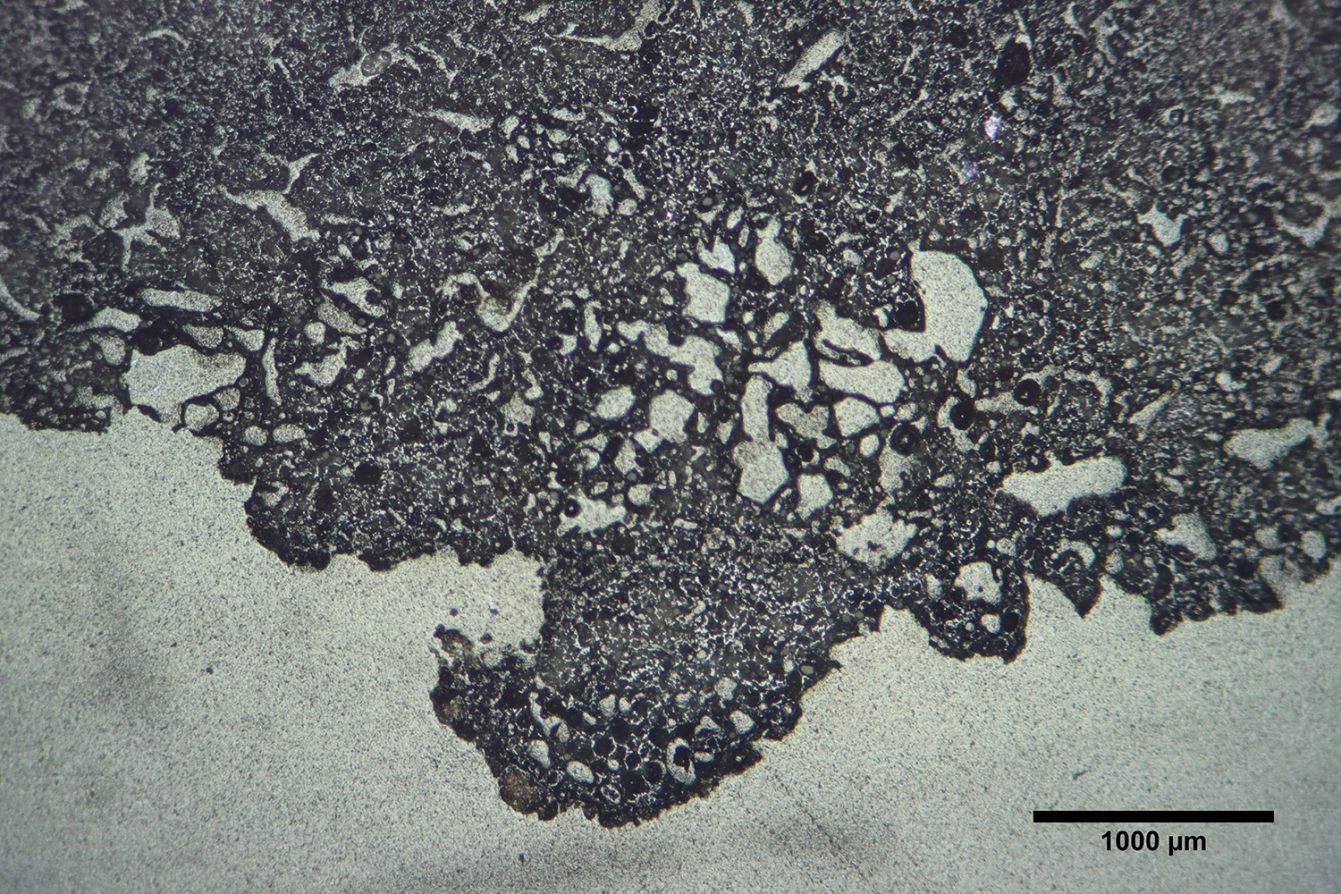
Vitrification of Mayapán tuyères. Photomicrograph of sample M-55 in reflected light demonstrating the bloated microstructure suggesting that the Mayapán blowpipe tips are heavily vitrified throughout. Photomicrograph by Jennifer Meanwell.

**Fig 6:**
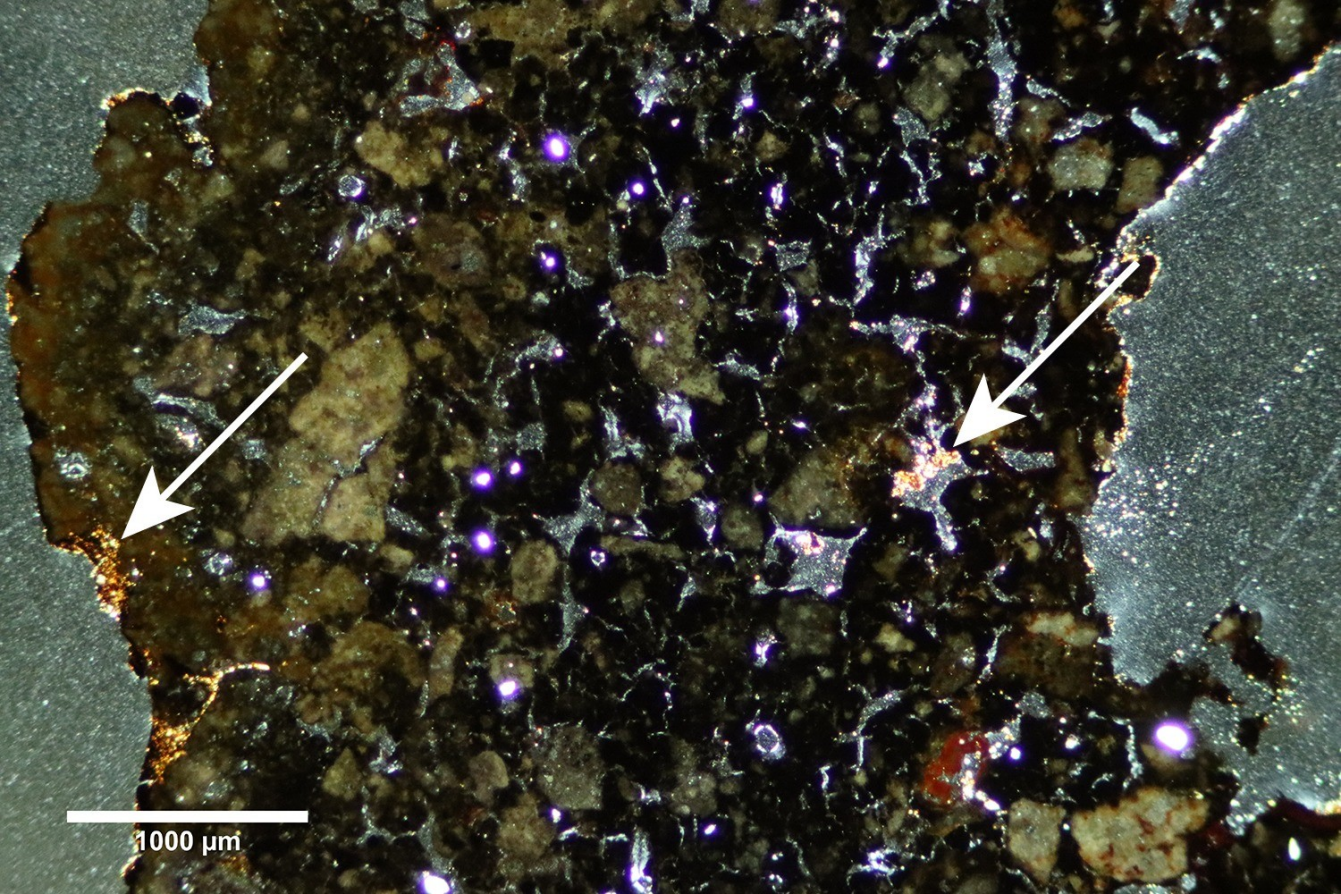
Photomicrograph of Mayapán tuyère paste. Photomicrograph in cross polarized light of M-55 with small amounts microcrystalline calcite (indicated by arrows) deposited along the exterior surface and in a few pores, interpreted as the result of post-depositional groundwater. The bright white crystalline inclusions are small pieces of quartz. Color variability suggest differential vitrification and highlights potential pieces of grog. Photomicrograph by Jennifer Meanwell.

**Fig 7:**
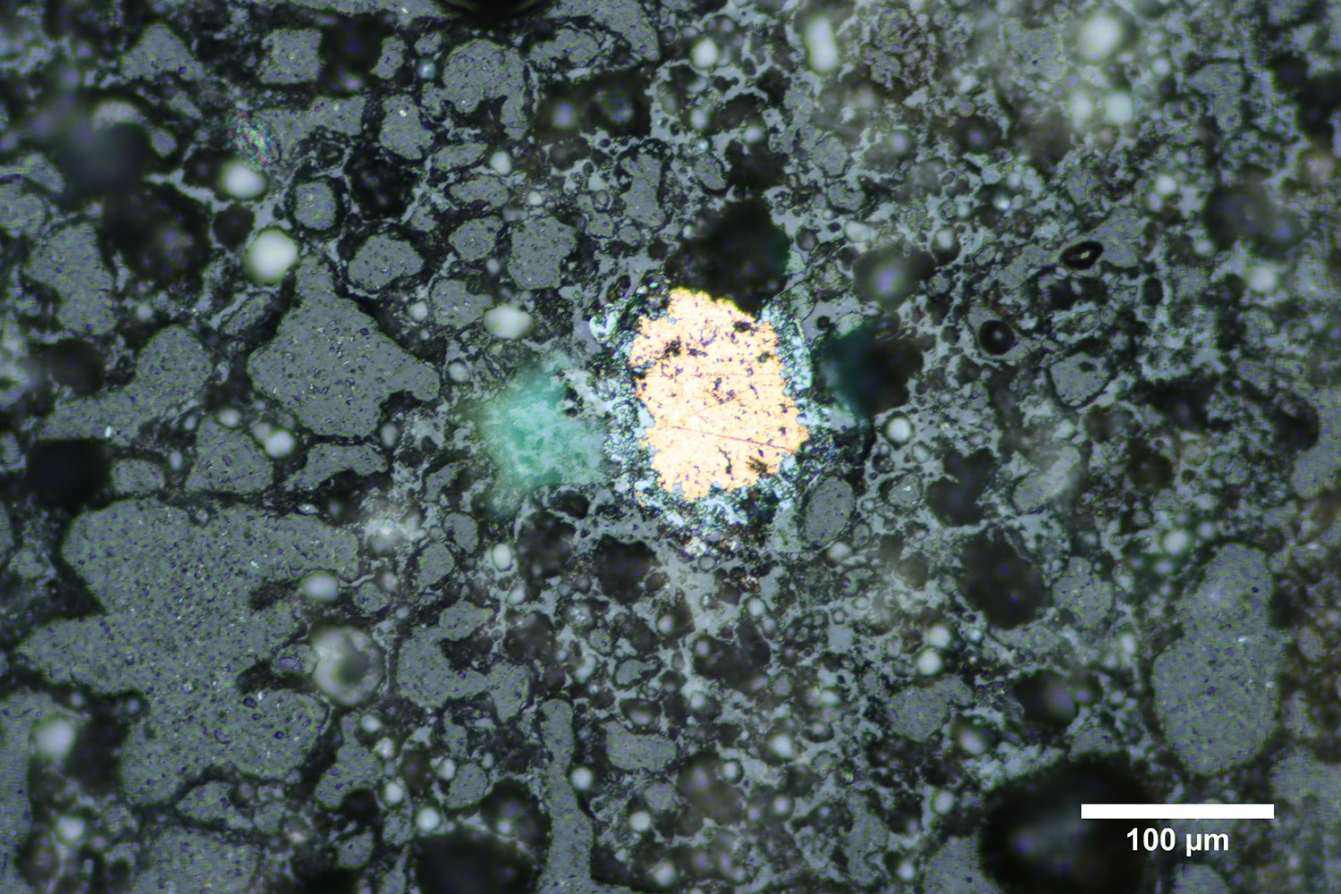
Photomicrograph of the copper prill in M-55. Photomicrograph of the copper prill within the walls of M-55. The copper color of the metal is visible in this reflected light image. Photomicrograph by Jennifer Meanwell.

**Fig 8:**
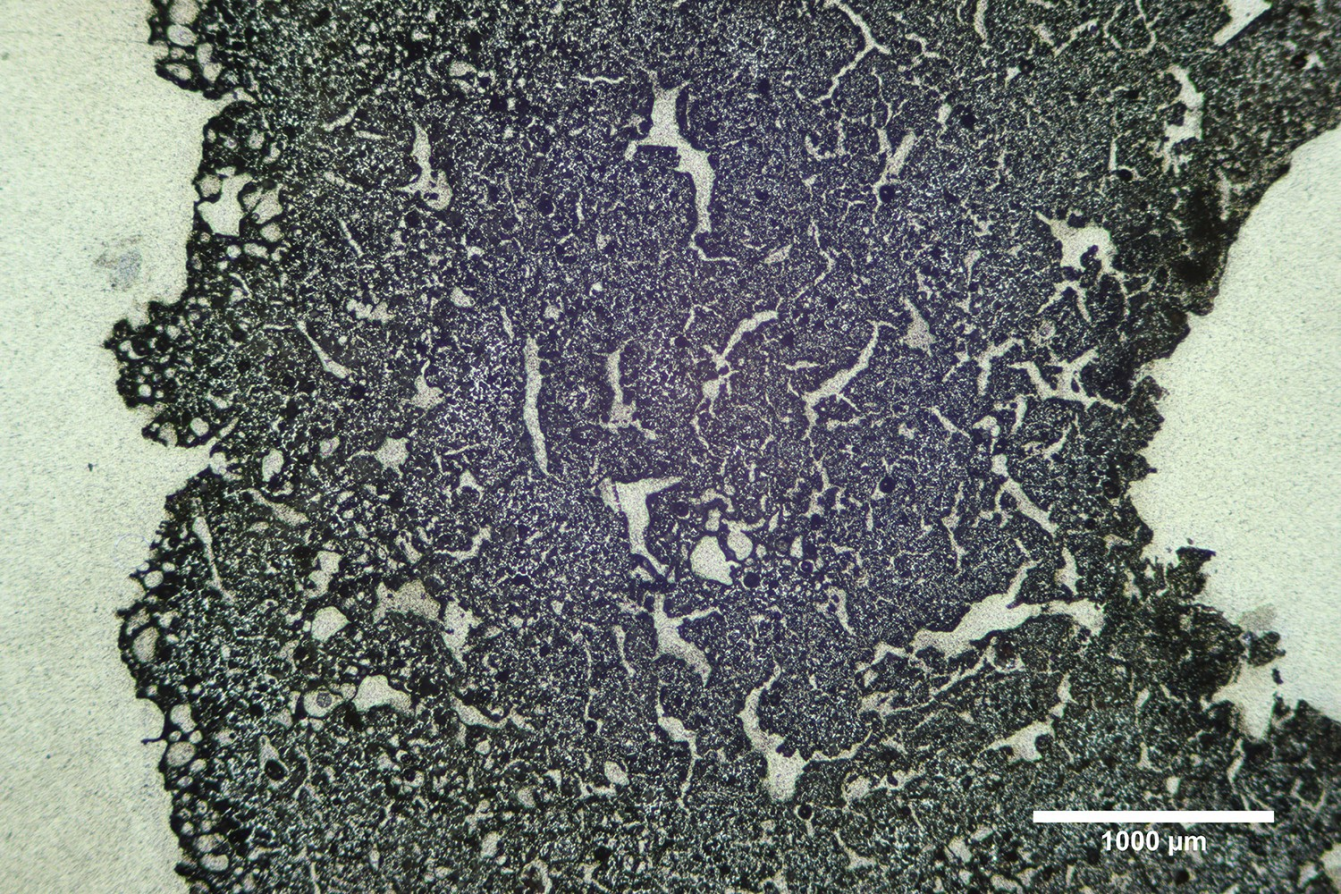
Photomicrograph of M-55 showing heat exposure on exterior. Photomicrograph of sample M-55 in reflected light showing the larger amount of vitrification and bloating along the exterior of the tip as compared to the area near the central hole. Photomicrograph by Jennifer Meanwell.

Petrographic analysis suggests that the pastes of the archaeological tuyères are heavily vitrified (Fig 5) and relatively free of mineral non-plastic inclusions, although small fragments of quartz are visible in a few locations (Fig 6). The pastes are also free of large calcite pieces, which are present in many local clay deposits from the local limestone bedrock. In a few cases, small amounts of microcrystalline calcite are found deposited into pores and cracks in the tips, but this is likely from post-depositional groundwater, rather than being part of the original paste (see Fig 6). Non-plastic inclusions help control shrinkage during drying and can increase thermal shock resistance via networks of small cracks; therefore, it seems unlikely that the Mayapán metallurgists were producing their metallurgical ceramics completely without non-plastic inclusions. Since we do not see many mineral inclusions, the most likely source of non-plastics is grog, or crushed fragments of previously fired material. Most local ceramics at Mayapán are full of calcite [7, 45], which breaks down at high temperatures, making these pastes not ideal for use as grog temper in metallurgical ceramics. Instead, it seems more likely that metallurgical ceramics were recycled as grog for the next set of blowpipe tips and crucibles. These potential grog fragments can be seen as areas of bloated and vitrified particles that are distinct from the rest of the matrix (Fig 6). One of the blowpipe tips (M-55) exhibits a copper prill in the middle of the ceramic paste (Figs 7 and 8), which was further analyzed via SEM-EDS to determine the composition of the prill.

### SEM-EDS analysis

**Fig 9:**
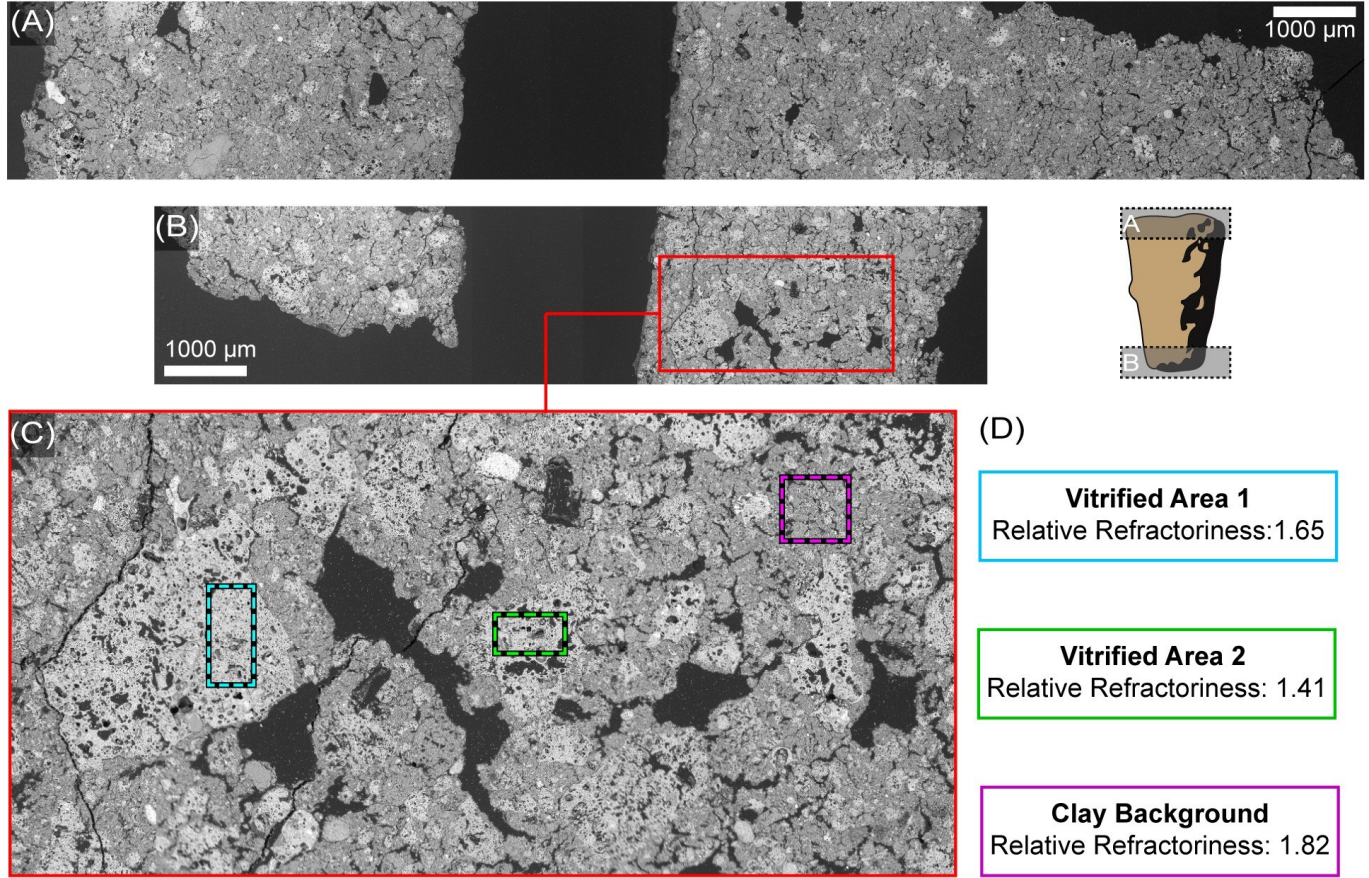
SEM-EDS Analysis of tuyère M-16. (A) Composite backscattered electron (BSE) micrograph along the hypothesized proximal of the tuyère showing frequent areas of increased vitrification, identified as brighter regions in the image. (B) Composite BSE micrograph along the hypothesized distal end of the tuyère, which exhibits more vitrification than the proximal end. (C) Higher magnification micrograph allows comparison of more vitrified regions (blue and green boxes) to the clay background (magenta box). (D) The relative refractoriness of each region in (C) calculated using quantified EDS data. Images by Jennifer Meanwell and Linda Seymour.

The archaeological tuyères show varying amounts of heat exposure both along the length of the fragments and through the thickness, with the outer surface and the small end of the tip most vitrified. Despite this gradient, the blowpipe tips are heavily vitrified throughout, even in the centers of the tips near their narrow holes (Fig 9A–9C). It is also clear that the broader and thicker end, hypothesized to be the proximal end of the piece (Fig 9A) is less vitrified than the smaller and thinner distal end (Fig 9B), suggesting less temperature exposure for the thicker end, which was presumably inside the blowpipe, than for the distal tip. Pieces of potential grog are also visible in both ends of M-16, which can be identified as distinct geometric particles of differential vitrification that stand out from the background clay matrix (Fig 9C).

To evaluate the potential that these areas of differential vitrification are due to chemical variation within the paste, chemical analysis (via EDS) was performed on a representative portion of tip M-16. These analyses, which are summarized as the relative refractoriness in Fig 9D and are reported in S1 Table, suggest that some of the extremely vitrified discrete fragments are chemically similar to the background paste in many cases, although analyses of other areas have identified occasional areas of more noticeable chemical difference, which would result in differential melting.

**Fig 10:**
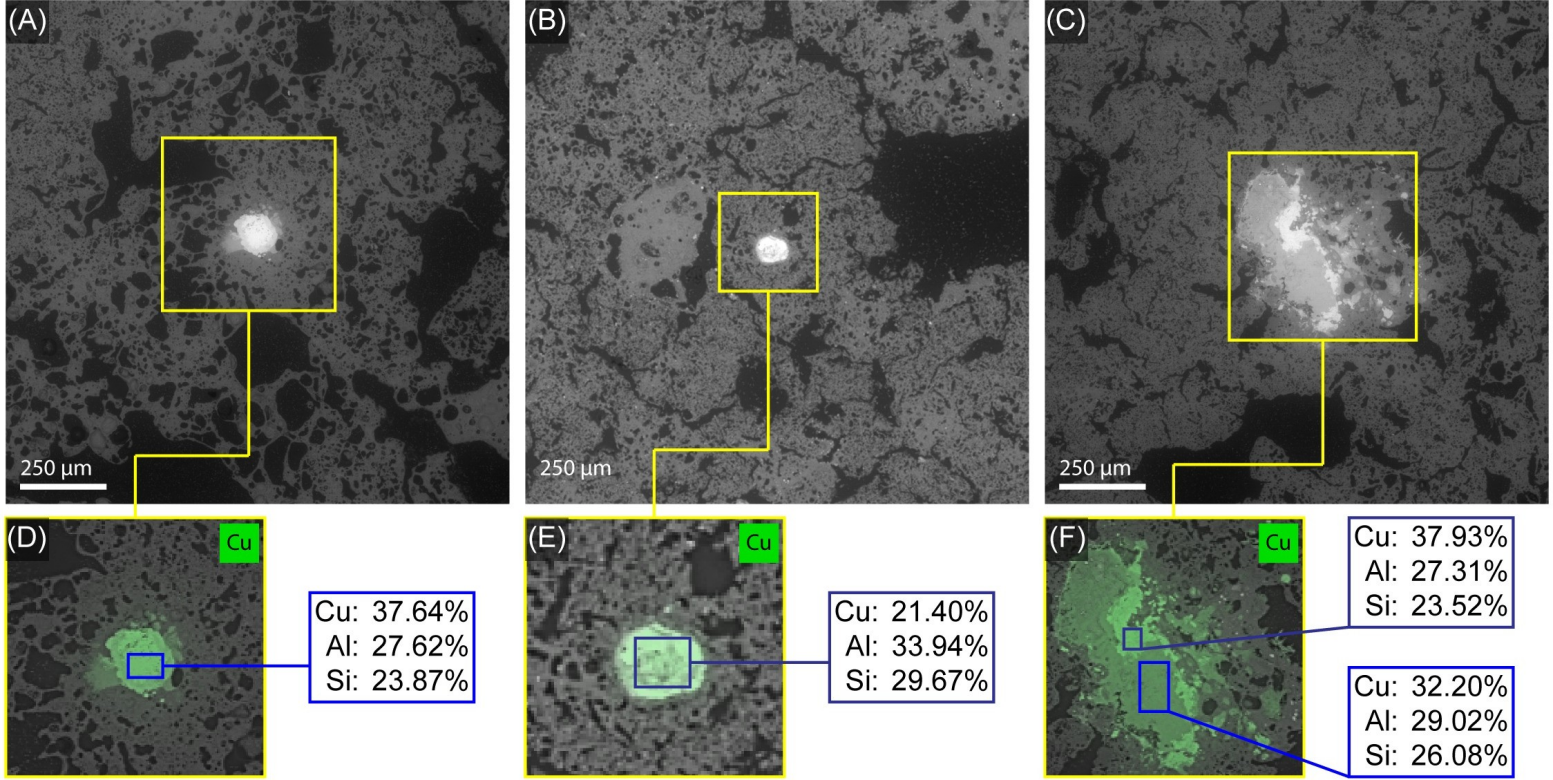
SEM-EDS Analysis of copper prills in tuyère M-55. (A-B) Backscattered electron (BSE) micrographs of copper prills. (C) BSE micrograph of copper-rich region in the tuyère. (D-F) Distribution of copper identified using EDS in the regions identified in (A-C) and quantified results (mass percent) of the three major elements identified. Images by Jennifer Meanwell and Linda Seymour.

The relative refractoriness of a paste can be evaluated using the ratio of aluminum, which is highly refractory, to the sum of alkali and alkaline earth elements and iron, which generally lower melting temperature [46]. In this type of evaluation, the more vitrified regions have lower refractory values, although the variation between vitrified and non-vitrified areas is small (Fig 9D). In both cases, the amount of calcium present is well below what is typically considered a “calcareous paste”, which contains upward of 6% calcium [47]. The values calculated for this material are higher (more refractory) than those found for Chinese bronze casting mold ceramics, which also needed to withstand high temperatures and which experimentally had melting temperatures in excess of 1200°C [46]. The small chemical variation present appears insufficient to cause the variability in vitrification levels observed. This suggests that the more vitrified areas (also found along surfaces of the vessels where they would be more directly exposed to heat) are pieces of grog from earlier molds and vessels that were crushed and added to the next generation of metallurgical ceramics. Grog also offers one explanation for the small copper prills (Figs 7 and 10) often found included in the metallurgical ceramics [7].

The blowpipe tips, like other metallurgical ceramics studied from Mayapán, also contain small copper-colored prills within the clay matrix. One large example is visible in M-55 (Figs 7 and 10A) and was previously discussed in the section on petrography and light microscopy. In addition, two other concentrations of copper were identified using SEM-EDS that were not easily identified in reflected light and had therefore been overlooked (Fig 10B and 10C). These include a small prill and a vitrified region of potential grog. As summarized in Fig 10D–10F, these copper-rich areas are predominantly a mixture of copper, silicon, and aluminum, with minor amounts of iron, calcium, and magnesium as reported in S2 Table. The specific chemistry of these areas is most likely due to an extended interaction time at high temperatures between a copper residue/prill and the surrounding aluminosilicate clay matrix, rather than an intentional mixture in the original metal work.

### Experimental study results

**Fig 11:**
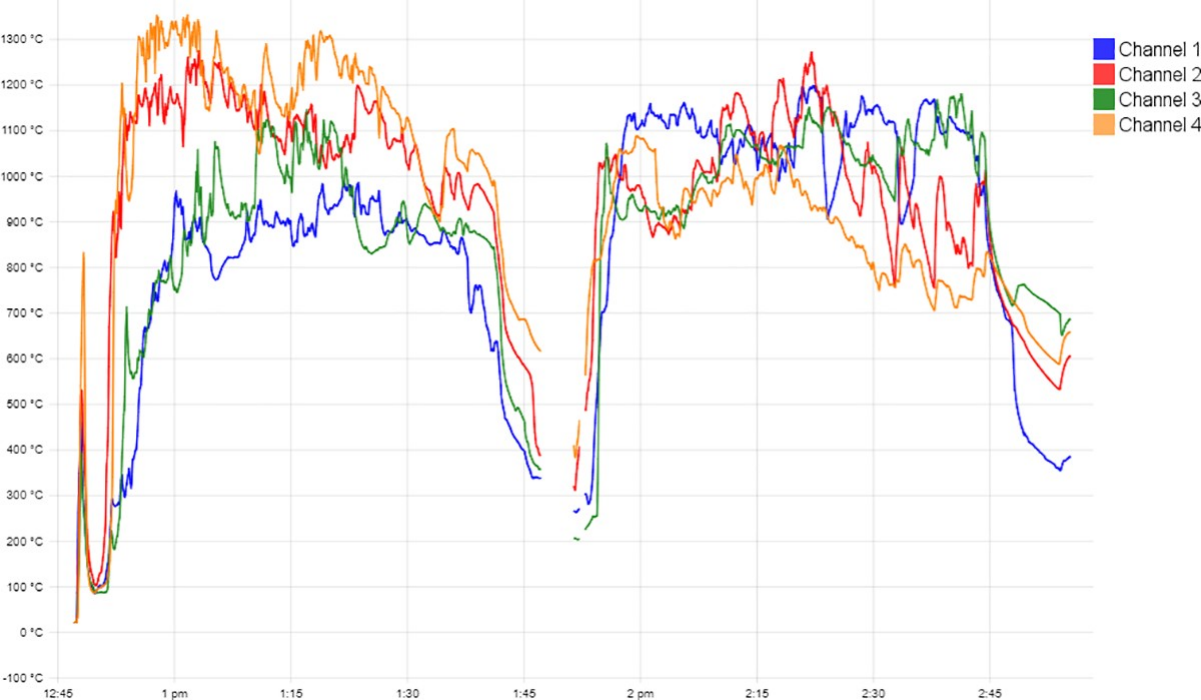
Temperatures reached using experimental tuyères. Results of the replication experiments with experimental tuyères, showing sustained temperatures above the melting point of copper. Temperatures from the thermocouples were recorded using PicoLog 6 software.

**Fig 12:**
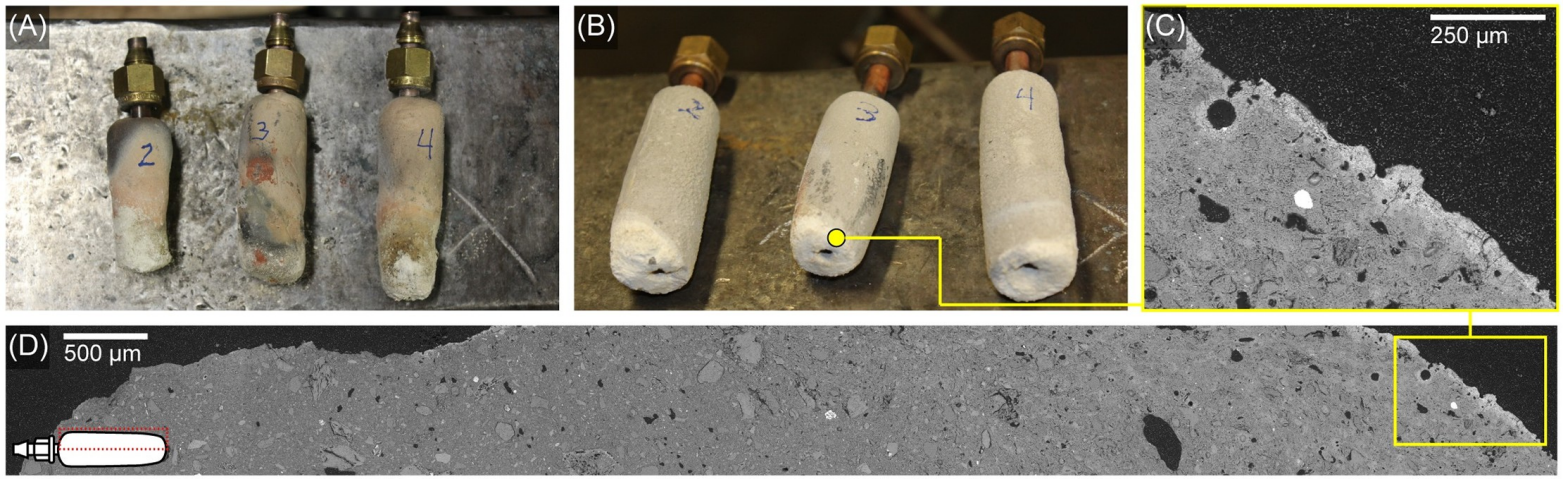
Experimental tuyères after firing test and SEM analysis. (A) Tuyères made from modern stoneware clay were heavily altered after firing and began to disintegrate at the distal ends closest to the heat source. (B) Yucatán white clay tuyères survived firing intact with minor alteration at the distal ends. (C) BSE micrograph of the experimental tuyère identified in (B) showing alteration along the external edge that was in direct contact with the heat source. (D) Composite BSE micrograph of the same experimental tuyère showing the vitrification is concentrated at the distal end. Photographs (A-B) by Elizabeth Paris. Micrographs (C-D) by Jennifer Meanwell.

In the replication experiments, the thermocouples placed within the larger crucible measured temperatures above the melting temperature of copper (1084°C), which were sustained for the length of the experiment once the high temperatures were reached (Fig 11). The temperatures were inconsistent across the large crucible interior, but the experimental tuyères were able to introduce enough air to keep the temperatures within the range needed for copper remelting for at least 20–30 minutes. If additional charcoal fuel had been added periodically, it is likely this temperature range could have been held indefinitely by using teams of blowpipe operators. The replica tuyères made from stoneware clay did not survive the firing experiment well (Fig 12A). The stoneware tips, rather than vitrifying, turned into fine alumina dust as the binding material burned out, since they did not reach the temperatures needed to vitrify. The local Yucatán white clay experimental tips, however, survived the replication experiment intact (Fig 12B), although they do show minor heat alteration especially in the narrow tip area that was closest to the flame.

## Discussion

### Blowguns and blowpipes

Blowguns were an ancient hunting technology that predated metalworking in the Maya area by many centuries. Blowguns were adapted to use as blowpipes within a metallurgical production context as the technology spread from West Mexico to various areas of Mesoamerica. Most scholars agree that Mesoamerican metallurgical production utilized blowpipe technology for both smelting and casting. The use of blowpipes is ethnohistorically documented for West and Central Mexico by several New World chroniclers [48, 49: pp. 69–78]. Its use in Tarascan metallurgy is attested through the depiction of metalworkers in the Lienzo de Jicalan [50: Fig 6] and the Relación de Michoacán [51: pl. XIX], and in Aztec metallurgy in the Florentine Codex [49: pp. 69–78] and the Codex Mendoza [52]. The images depict the metalworkers seated around small, above-ground furnaces (annular-base bowls in the Tarascan sources; large, flat-base tripod dishes in the Aztec sources), presumably made of ceramic or stone, blowing into their blowpipes, and surrounded by the products of their labor [21, 50].

The use of blowpipes may be inferred for the K’iche’ highlands, from a ceramic vessel depicting blowpipe use, in association with a metalworking production area. The vessel is a Fortress White-on-Red globular jar recovered at the Late Postclassic period site of El Resguardo, part of Greater Utatlán [39: pp. 64]. The vessel is decorated with a modeled figure, seated with elbows on knees, with a thick shaft extending from its mouth to the vessel body wall. The figure wears a tri-lobed headdress, and a long, painted tail on the vessel wall extends from the rump of the figure, suggesting that it represents a monkey, the patron deity of artisans and crafters in K’iche’ religious beliefs [39]. Furthermore, the vessel was recovered near the south balustrade of a structure where 26 ceramic ingot molds were recovered, further supporting the interpretation of the vessel as depicting a monkey patron deity of metalworkers [38, 39].

We suggest that the technology for manufacturing metalworking blowpipes may be similar to, and possibly derived from, the technology for blowgun manufacture in pre-Columbian Mesoamerica. Blowguns are long, hollow, and wooden instruments, used as weapons in both hunting and warfare in many areas of the world [53–55]. Historically, their use was particularly documented in tropical and semi-tropical areas of Mesoamerica, Amazonia, and the Gulf Coast including the Southeastern US [53], where they were primarily used to hunt birds and small animals [56]. Blowguns were in widespread use from Central Mexico to the Yucatán Peninsula, as documented at Spanish Contact by numerous observers, including Fernando Cortés [57, 58], Bernal Díaz del Castillo [59], Fray Bernardo de Sahagún [60], and Fray Diego de Landa [61], which has been reviewed in detail by Ventura [56]. Blowguns were commonly used into the mid-20^th^ century; in the 1930’s the Guatemalan government outlawed their use to protect small game, particularly Guatemala’s national bird, the quetzal [56].

The widespread use of the clay pellet blowgun is attested by its use in the creation myths of numerous Maya communities including the K’iche’ and Kekchi, as well as those of the Zapotec of Mitla, Oaxaca [62]. It is particularly central to the Popol Vuh of the K’iche’ Maya, where it is the weapon of the Hero Twins, Hunahpu and Xbalanque, who use it to hunt birds for food, injure the False Sun (Vucub Caquix or 7 Macaw), trick their abusive brothers, and hide inside of it during their trials in Xibalba, the underworld [63]. Although blowguns made of wood generally do not survive in tropical or neotropical environments, they are depicted on over 25 Classic period pre-Columbian Maya ceramic vessels, often in mythological scenes featuring the Hero Twins [56]. A vessel fragment from Teotihuacan also depicts a man hunting birds with a blowgun, suggesting that its use was widespread throughout pre-Columbian Mesoamerica [56].

Ventura’s review [56] of blowgun production techniques in southeast Mesoamerica suggests several different techniques for the production of blowguns. Branches or saplings of many different tree species are used, including the mogotillo tree (*Saurauia englesingii*; Jicaque culture, Honduras), the white pine (*Pinus spp*., Mam Maya, western Guatemala), *moquillo* and *ixkepatze* (Concepción Tutuapa, Department of San Marcos, Guatemala), and *ol ch’am* (*Saurauia spp*., Jacaltenango, western Guatemala*)*. Southeast US indigenous groups such as the Catawba and Cherokee, also used river cane [64]. Importantly, many or most of these species have a soft core which can be more easily removed to create the hollow tube for the blowgun. Some cultures, such as the Mam Maya, split the branch in half in order to hollow out the center, later adhering the two halves with glue or nails [65], while other groups such as the Jicaque and Jacaltec Maya hollowed out the entire pith using a vine or wire, such that the instrument is of a single piece. In some cases, different techniques were used to soften or rot the soft core, such as heating the wood over a fire, or submerging the end of the branch in water [56].

Ethnographically-documented blowguns range significantly in size. Southeast Mesoamerican blowguns range significantly in length, with documented examples from 1.19 m (K’ekchi’ Maya, Belize) to 2.5 m (Jicaque, Honduras). Ventura’s [56] Jacaltec Maya informants claimed that the best blowgun is long and relatively heavy, to provide better stability for good aim. During their own ethnographic study of blowgun use in Jacaltenango, La Farge and Byers [66] observed that a blowgun was ideally 50 cm longer than the height of the individual using it. The inner diameter of the core was about 1.3–1.4 cm for Jacaltec blowguns, smoothed with a wire wrapped in sisal (henequen, *ixtle*) twine. The exterior of the blowgun was stripped of bark, and smoothed with sandpaper, with an exterior diameter of approximately 3.5 to 4 cm, and a carved mouthpiece [56]. When fitted with a clay blowpipe tip, implements of these dimensions would have been highly suitable for metalworking, allowing metalworkers to remain at a safe distance from the furnace while supplying it with a steady airflow. These dimensions of the Mayapán tuyères are highly consistent with the 1.3–1.4 cm inner diameters of Jacaltec Maya blowguns: The external diameter of the blowpipe tip from Q-40a (M-16) measured 1.6 cm at the proximal end, tapering to 1 cm at the distal end; while the tip from Q-99 (M-55) measured 1.4 cm at the proximal end, tapering to 1 cm at the distal end.

### Blowpipe tips in comparative analysis

Blowpipe tips or tuyères are not commonly reported archaeological objects, even at sites with known metallurgical traditions. Therefore, scholarly understanding of the parameters defining functional or high-performance blowpipe tips are not well established. Here, we discuss the known archaeological tuyères that were potentially used in small-scale metallurgy, similar to that of Mayapán. Furnaces at Batane Grande in Peru were associated with ceramic-tipped blowpipes. The reported internal diameters of the ceramic tips were relatively uniform at 8 +/- 1 mm; these were used for the purpose of smelting ore in a bowl furnace [67]. These tip apertures are much larger than the Mayapán examples. Rehder [43] suggests that in order to obtain adequate penetration of breath into the fuel bed, the internal diameter of ceramic blowpipe nozzles must be kept small, limited to a diameter of about 5–10 mm. He notes that small nozzles may be considered diagnostic for the use of blowpipes, relative to the much larger sizes of typical apertures for ceramic tuyères fed by bellows [43].

Del Pino Curbelo and colleagues [68] report tuyères from the site of Las Pilas in Almeria, Spain, where the earliest evidence of metalworking dates to the beginning of the third millennium BC. They report a small blowpipe tip 7.6 cm long and 4.1 cm in diameter, with a tapered aperture approximately 7 mm. As at Mayapán, Ca-poor clays were used in metallurgical ceramics, although notably, similar clays were also chosen for domestic pottery used primarily as roasting pots for grain.

Day and colleagues [69] also reported two small tuyères or blowpipe tips, from the site of Poros Katsambas, located on the north coast of Crete, downriver from the Minoan political capital of Knossos. The tuyères were associated with numerous other artifacts of metallurgical production such as crucibles, slag, dross, and moulds for mid-rib daggers [69, 70]. Notably, the tuyères were much larger than those of Mayapán; the smaller tuyère had a terminal aperture of 7 mm and a wall thickness of 6 mm; while the larger tuyère had a terminal aperture of 44 mm and a wall thickness of 19 mm; both tuyères exhibited vitrification on the terminal end. Doonan and colleagues [70] suggest that the smaller tuyère was associated with work that required careful localized heating, while the larger tuyère was used for less delicate work. The dimensions of the Mayapán tuyères are much smaller than both of these examples, suggesting that they were used for the fine work that would have been associated with the delicate copper bells and finger rings that make up the bulk of its metal artifact assemblage.

### The Mayapán tuyères and local metalworking practice

Although we cannot fully reconstruct the *chaîne opératoire* for the tuyères from Mayapán, a partial process can be summarized. The craftspersons making the tuyères either selected a clay deposit with very little included calcite (unlikely, given the geologic context of the northern Yucatán), or processed the clays to remove as much calcite as possible. Then, the clay was mixed with grog made from earlier metallurgical ceramics, which would occasionally introduce prills of copper. The ceramics, including the tuyères presented here and the crucibles and molds previously studied, were designed and produced at a small scale that fit with the small amounts of metal being used, the small size of the objects being produced, and the traditional interior diameters of Maya blowguns or blowpipes [2, 7, 35]. These tuyères have smaller apertures than other documented tuyères from other metallurgical traditions [67–70], providing tightly focused air to specific points in the furnace, allowing localized temperature adjustments. The experimental replication studies we performed are vital to allowing us to suggest that these tiny artifacts are functional tuyères.

The production techniques utilized by the Mayapán metallurgists are highly influenced by the local environment and pottery traditions. Grog worked well as a non-plastic material given the extremely calcareous environment, and may have partially mitigated the laborious preparation of highly-calcareous local clays. Grog was also a known tempering agent used in cooking and storage vessels at Mayapán, perhaps making it easier for the artisans making metallurgical ceramics to adopt this familiar technique [7]. We cannot say with specificity that grog is ideal for metallurgical ceramics produced elsewhere with easier access to different raw materials, and this issue, raised by an anonymous reviewer, merits further investigation through experimental study and/or petrographic analysis of tuyères from other world areas.

These objects may perhaps serve as a cautionary tale for archaeologists and materials scientists who study metallurgy and ceramics. The clay calcium-reduction step outlined above alters the overall chemistry of the surrounding clays, which could distort a bulk chemical signature for these artifacts as compared to other vessels from Mayapán or to the local raw materials deposits. Also, the Mayapán artisans produced tools that just barely functioned for their intended purpose. These are not modern refractory ceramics that can survive very high temperatures. Indeed, the characteristic vitrification and bloating that allowed these ceramics to be initially identified suggests that they are functioning very close to their melting point. Therefore, it is always important to perform technical analyses of tools from the specific tradition under study. Although materials constraints are real and do affect production choices, there are multiple ways to solve these engineering challenges, and archaeologists must not assume that the solutions from one time and place are broadly applicable.

## Conclusions

The ceramic tuyères documented here are the first known examples of this item from Mesoamerica. Although we cannot be certain that these items were used as tuyères, the external dimensions, size of internal perforations, high degree of vitrification, and the presence of copper prills in the ceramic fabric, suggest that they were used in pyrotechnological production. The combined results are consistent with the previous evidence for metalworking at Mayapán [2, 7, 35]. The replication experiments reported in this study suggest that when combined with wooden blowpipes, the Mayapán tuyères would have been sufficient for small-scale, furnace-based metallurgy, of the type identified at Mayapán during the Postclassic period. Other metallurgical ceramics from the site, such as crucibles and molds, are also very small and appear to have been designed specifically for the small volumes of metal that were being imported, remelted, and formed into bells and other decorative objects [7].

Mayapán’s position within an extensive political and economic network in Postclassic period Mesoamerica allowed its artisans access to raw materials and knowledge that were integrated into their specific metallurgical community of practice. Ancient metallurgists at Mayapán adapted and remastered several linked crafts, including the metallurgy itself, blowpipe technology, and metallurgical ceramics with heat-resistant properties required for functional crucibles and tuyères. Additionally, they engineered their metallurgical ceramics to survive within a high temperature environment by processing out the large crystalline calcite present in many local clay deposits and integrating grog from previous metallurgical ceramics. However, they did not “over-engineer” these ceramics. Although they function at the temperatures required to melt copper, the extremely vitrified and bloated pastes suggest that these ceramics were functioning close to their melting point, and likely would not be considered “refractory” in the modern sense of the word.

The discovery of tuyères at Mayapán also sheds light on the ways in which communities of practice adapt non-local high-skill crafting technologies to local contexts and materials. At Mayapán, tuyères and other metallurgical ceramics were made from local materials, in local styles that adapted traditional blowgun parameters, grog-based clay fabric preparations, and the widespread availability of high-quality beeswax for castings; other technological aspects of the process were most likely adapted from distant production zones. This is particularly significant for the Maya region, in which the products of high-skill technologies are often attributed to itinerant craftspersons or traveling merchants, assumed to have superior knowledge and ability than local artisans [34], reinforcing outdated ideas of the Postclassic period Maya as a post-collapse, “decadent” society [71]. The discovery of some metalworking paraphernalia in a colonnaded hall within the monumental zone and an adjacent crafts barrio suggests that high-skill craftspersons were not isolated iconoclasts, but that many of the city’s metalworkers were patronized by the powerful rulers of the Mayapán confederacy [72, 73]. The elaboration of a highly localized metalworking tradition likely served as an important source of wealth and status, reinforcing the city’s image as a cosmopolitan core center within the broader political and economic networks of the Postclassic Mesoamerican world.

## Supporting information

S1 Table EDS analysis of M-16. Average chemical composition (at%) of three areas within M-16 measured by EDS as well as the relative refractoriness (Al/K+Na+Ca+Mg+Fe).
(DOCX)

S2 Table EDS analysis of prills in M-55. Average chemical composition (at%) of four copper-rich areas within M-55 measured by EDS.
(DOCX)

S1 Fig EDS spectrum of area 1 of M-16. Graph of the energy counts for area 1 in M-16 as depicted in Fig 9C. K_α_ energy lines are also marked.
(TIF)

S2 Fig EDS spectrum of area 2 of M-16. Graph of the energy counts for area 2 in M-16 as depicted in Fig 9C. K_α_ energy lines are also marked.
(TIF)

S3 Fig EDS spectrum of area 3 of M-16. Graph of the energy counts for area 3 in M-16 as depicted in Fig 9C. K_α_ energy lines are also marked.
(TIF)

S4 Fig EDS spectrum of copper prill in M-55. Graph of the energy counts for the copper prill in M-55 as depicted in Fig 10A and 10D. K_α_ energy lines are also marked.
(TIF)

S5 Fig EDS spectrum of small copper prill in M-55. Graph of the energy counts for the small copper prill in M-55 as depicted in Fig 10B and 10E. K_α_ energy lines are also marked.
(TIF)

S6 Fig EDS spectrum of copper-rich area in M-55. Graph of the energy counts for a copper-rich area in M-55 as depicted in Fig 10C and 10F. K_α_ energy lines are also marked.
(TIF)
