# Supplementary material for: Blowpipes and their metalworking applications: New evidence from Mayapán, Yucatán, Mexico
Source: PLoS One. 2020 Sep 22;15(9):e0238885. doi: 10.1371/journal.pone.0238885 (PMC7508413; doi:10.1371/journal.pone.0238885)
Supplement: S1 Table — Average chemical composition (at%) of three areas within M-16 measured by EDS as well as the relative refractoriness (Al/K+Na+Ca+Mg+Fe). (DOCX) [file pone.0238885.s001.docx]

**S1 Table. EDS analysis of M-16.**

| **Element** | **Area 1 (Vitrified)** | **Area 2 (Clay Background)** | **Area 3 (Vitrified)** |
| --- | --- | --- | --- |
| **Cl (in epoxy)** | 0.08 | 0.16 | 0.13 |
| **K** | 0.79 | 0.55 | 0.72 |
| **Al** | 11.99 | 9.07 | 10.10 |
| **Si** | 12.91 | 8.79 | 10.04 |
| **Na** | 0.20 | 0.12 | 0.19 |
| **Ca** | 2.21 | 1.53 | 2.69 |
| **Fe** | 2.90 | 2.06 | 2.51 |
| **Mg** | 1.15 | 0.70 | 1.02 |
| **Ti** | 0.30 | 0.28 | 0.26 |
| **Relative Refractoriness** | 1.65 | 1.82 | 1.41 |

Average chemical composition (at%) of three areas within M-16 measured by EDS as well as the relative refractoriness (Al/K+Na+Ca+Mg+Fe).
