# Supplementary material for: Blowpipes and their metalworking applications: New evidence from Mayapán, Yucatán, Mexico
Source: PLoS One. 2020 Sep 22;15(9):e0238885. doi: 10.1371/journal.pone.0238885 (PMC7508413; doi:10.1371/journal.pone.0238885)
Supplement: S2 Table — Average chemical composition (at%) of four copper-rich areas within M-55 measured by EDS. (DOCX) [file pone.0238885.s002.docx]

**S2 Table. EDS analysis of prills in M-55.**

| Element | Large Prill | Small Prill | Area 1 of Copper-rich area | Area 2 of Copper-rich area |
| --- | --- | --- | --- | --- |
| Cu | 37.64 | 21.4 | 37.93 | 32.2 |
| Al | 27.62 | 33.94 | 27.31 | 29.02 |
| Si | 23.87 | 29.67 | 23.52 | 26.08 |
| Fe | 3.4 | 5.32 | 3.54 | 4.02 |
| Ca | 2.71 | 3.93 | 2.39 | 3.22 |
| Mg | 2.76 | 2.91 | 3.48 | 3.12 |
| K | 0.94 | 1.48 | 0.8 | 1.17 |
| Cl | 1.06 | 0.9 | 0.91 | 0.71 |
| P | 0 | 0.45 | 0.12 | 0.45 |

Average chemical composition (at%) of four copper-rich areas within M-55 measured by EDS.
