# Supplementary figures and images for: Blowpipes and their metalworking applications: New evidence from Mayapán, Yucatán, Mexico
Source: PLoS One. 2020 Sep 22;15(9):e0238885. doi: 10.1371/journal.pone.0238885 (PMC7508413; doi:10.1371/journal.pone.0238885)

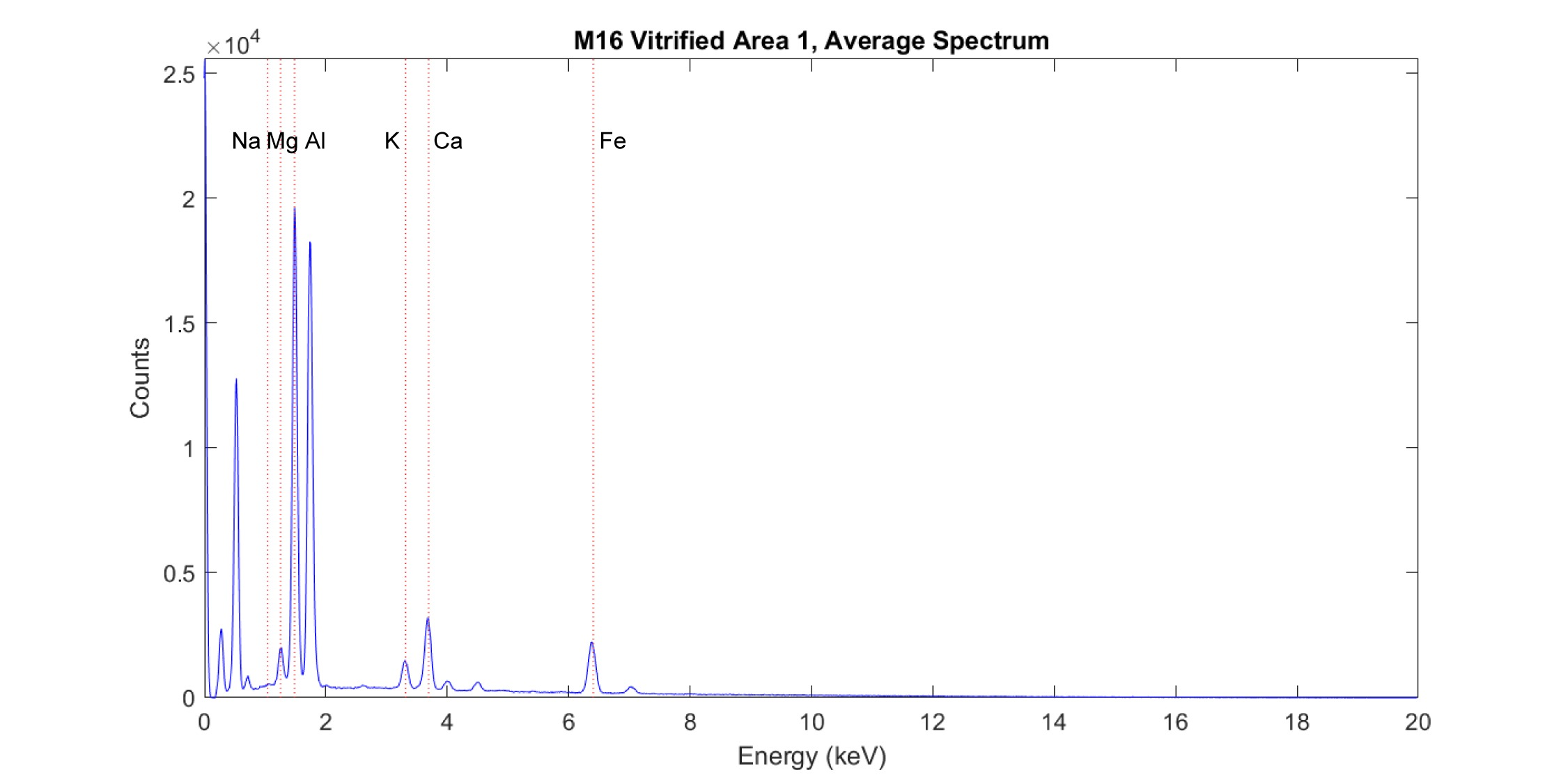

Supplement: S1 Fig — Graph of the energy counts for area 1 in M-16 as depicted in Fig 9C. Kα energy lines are also marked. (TIF) [file pone.0238885.s003.tif]

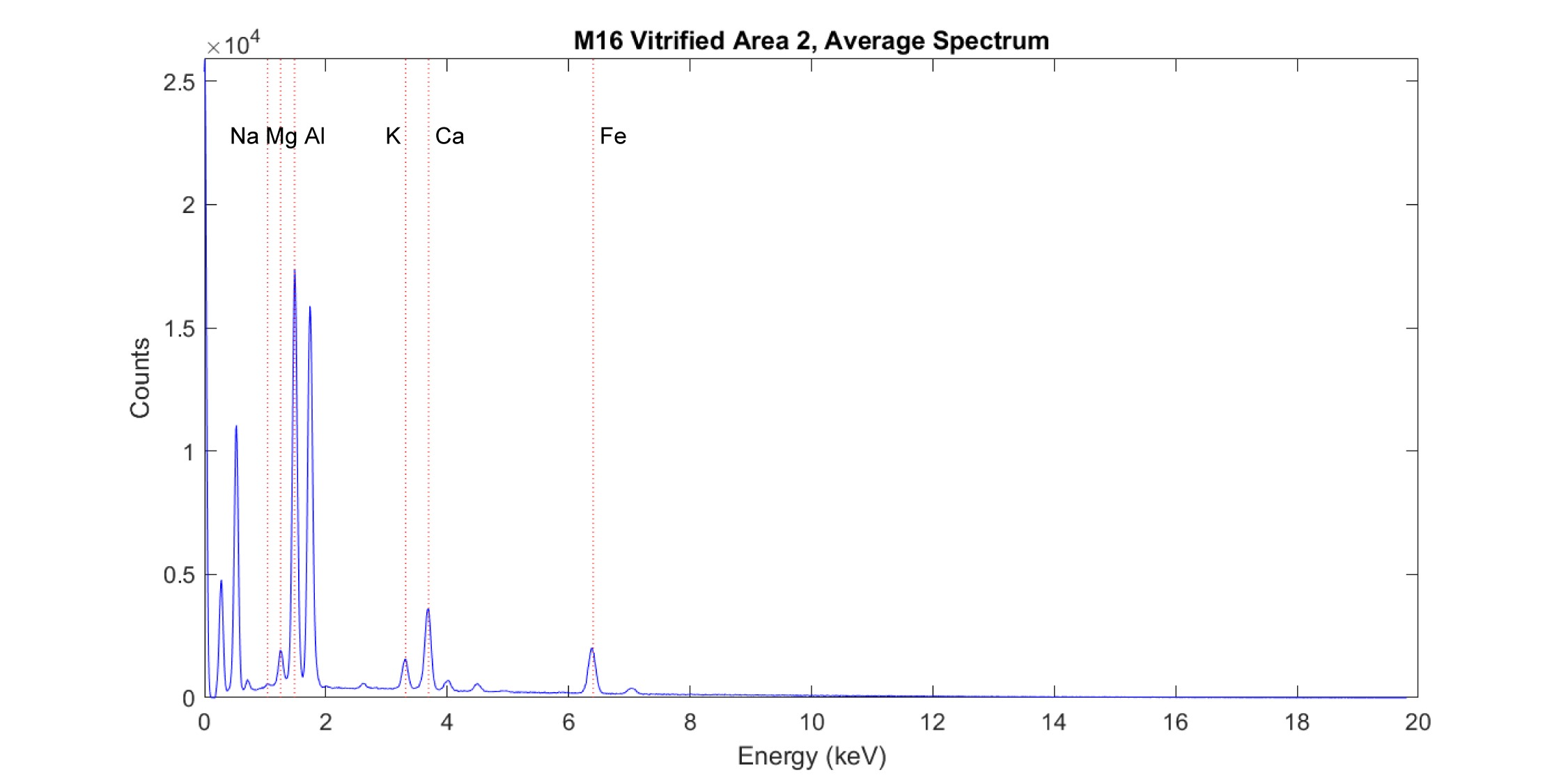

Supplement: S2 Fig — Graph of the energy counts for area 2 in M-16 as depicted in Fig 9C. Kα energy lines are also marked. (TIF) [file pone.0238885.s004.tif]

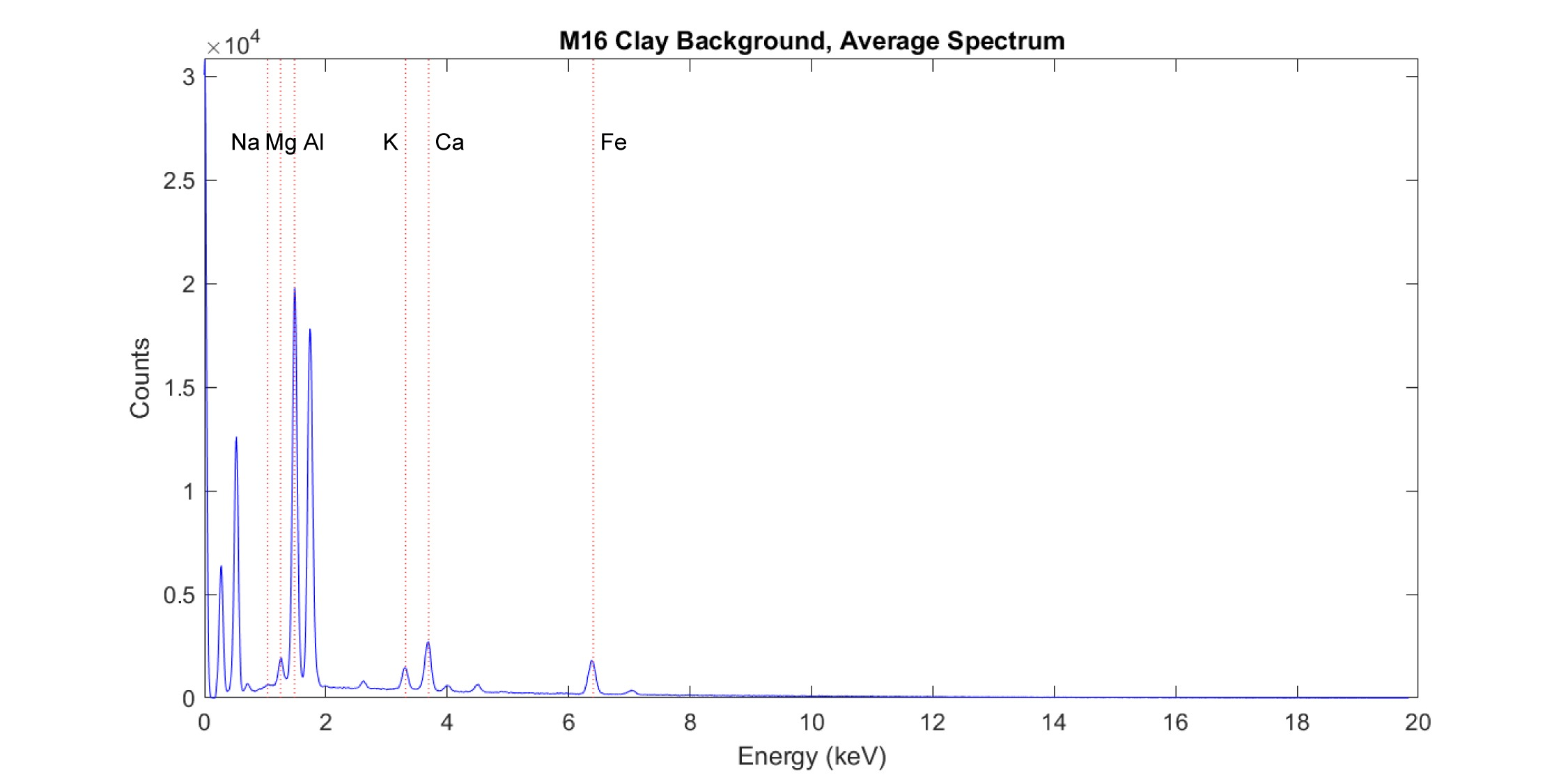

Supplement: S3 Fig — Graph of the energy counts for area 3 in M-16 as depicted in Fig 9C. Kα energy lines are also marked. (TIF) [file pone.0238885.s005.tif]

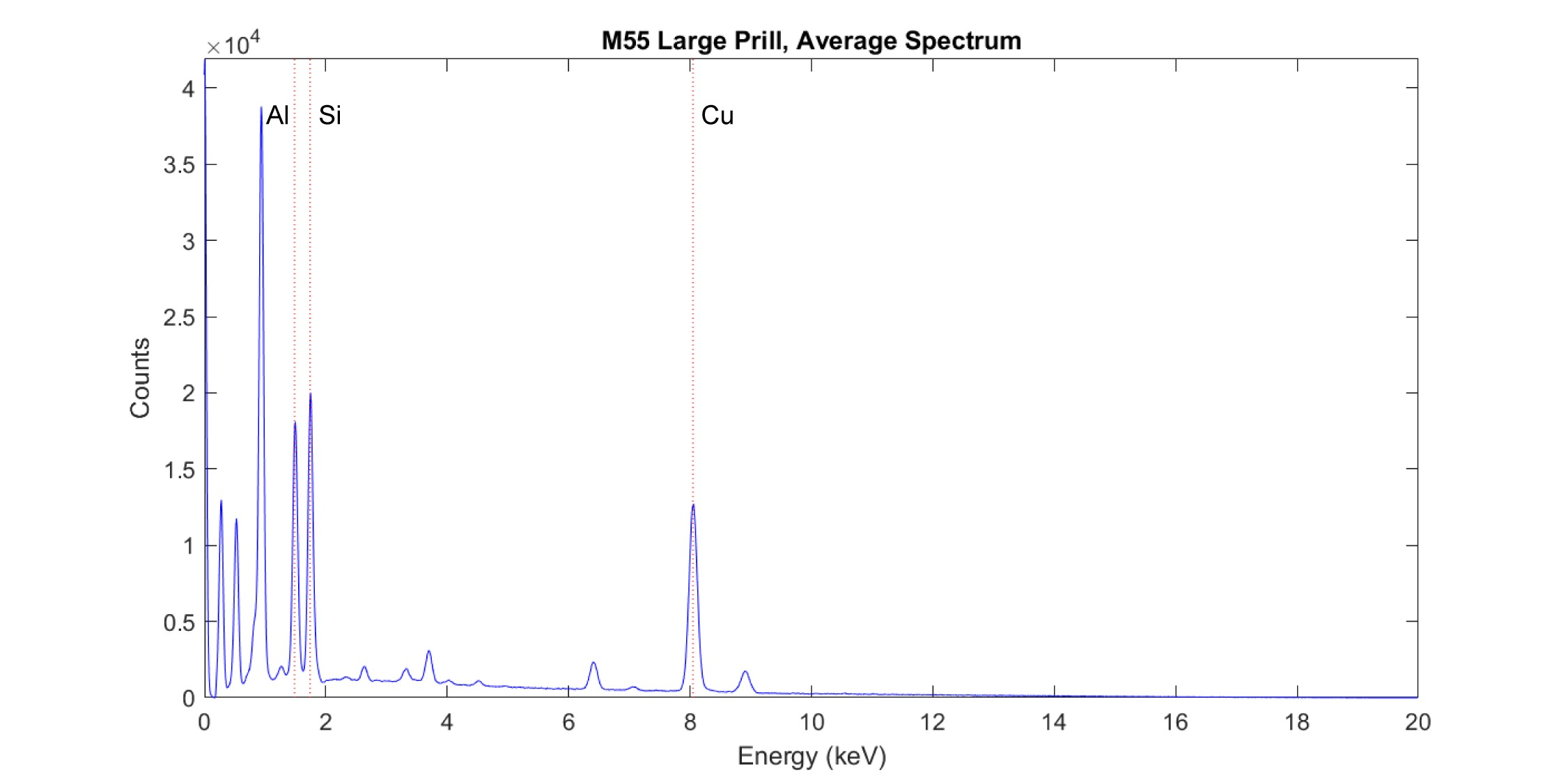

Supplement: S4 Fig — Graph of the energy counts for the copper prill in M-55 as depicted in Fig 10A and 10D. Kα energy lines are also marked. (TIF) [file pone.0238885.s006.tif]

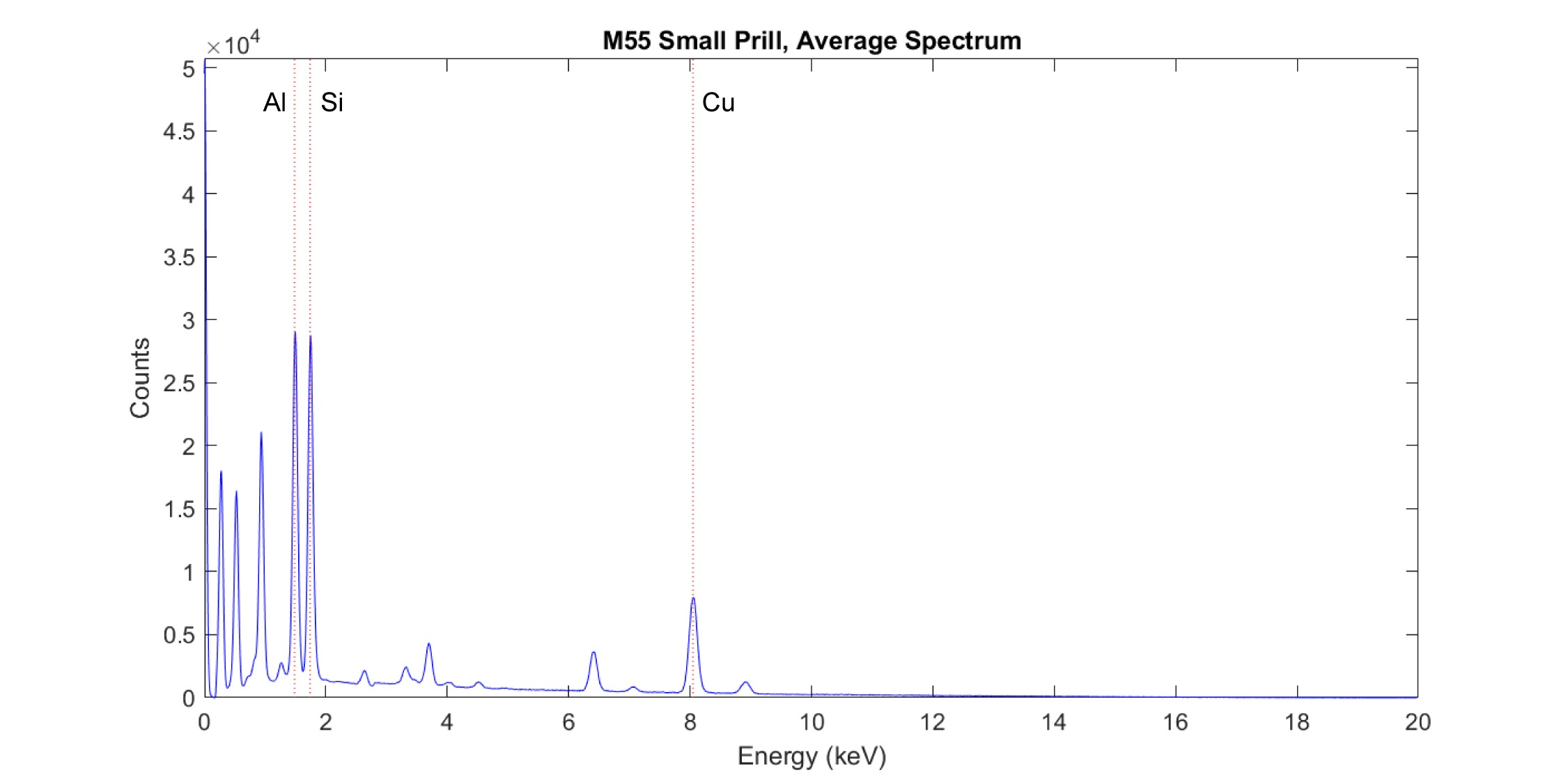

Supplement: S5 Fig — Graph of the energy counts for the small copper prill in M-55 as depicted in Fig 10B and 10E. Kα energy lines are also marked. (TIF) [file pone.0238885.s007.tif]

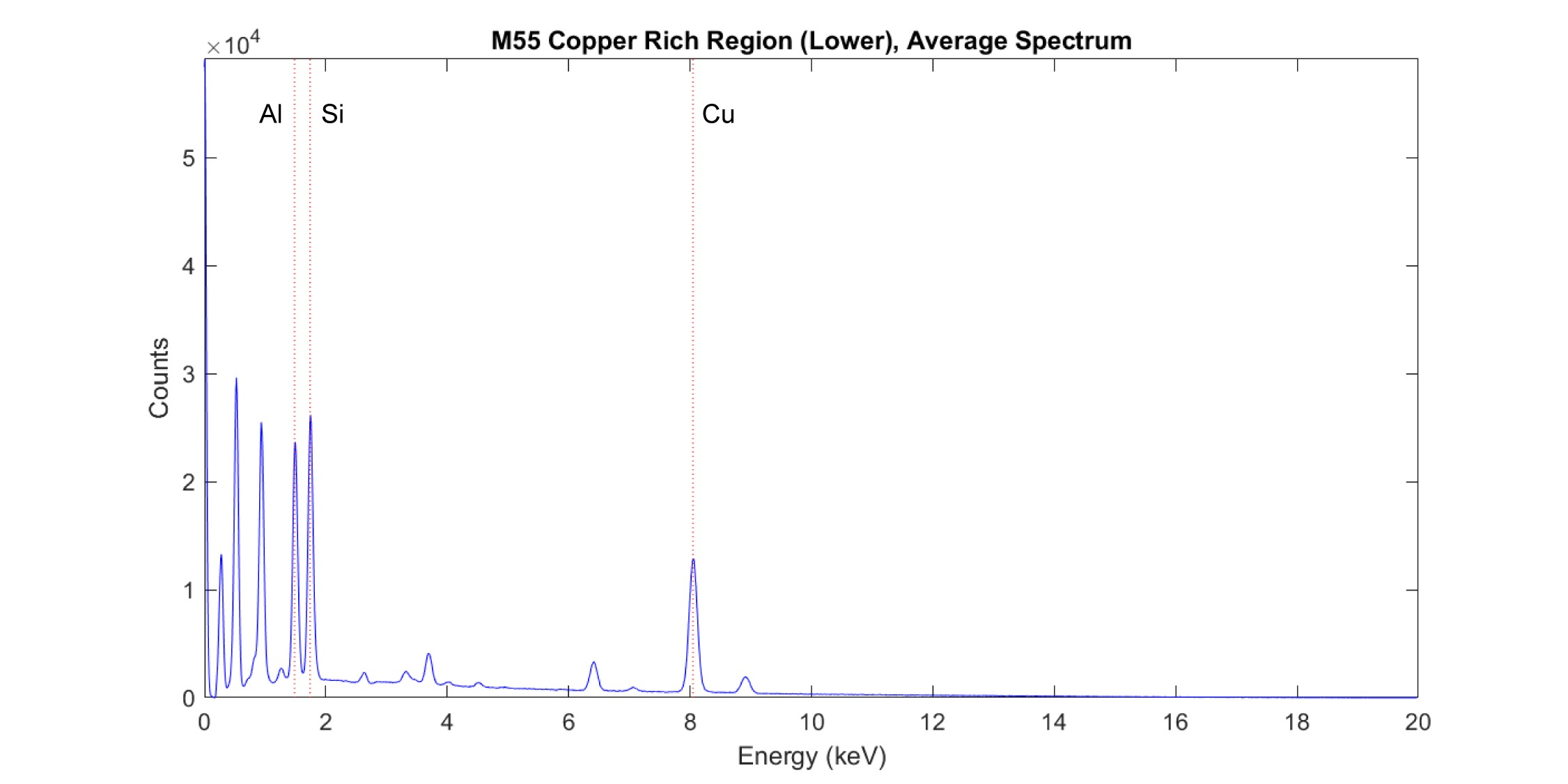

Supplement: S6 Fig — Graph of the energy counts for a copper-rich area in M-55 as depicted in Fig 10C and 10F. Kα energy lines are also marked. (TIF) [file pone.0238885.s008.tif]
